# Metabolic engineering of *Saccharomyces cerevisiae *for the production of n-butanol

**DOI:** 10.1186/1475-2859-7-36

**Published:** 2008-12-03

**Authors:** Eric J Steen, Rossana Chan, Nilu Prasad, Samuel Myers, Christopher J Petzold, Alyssa Redding, Mario Ouellet, Jay D Keasling

**Affiliations:** 1 Joint BioEnergy Institute, 5885 Hollis Avenue, Emeryville, CA 94608, USA; 2 Department of Bioengineering, University of California, Berkeley, CA 94720, USA; 3 Physical Biosciences Division, Lawrence Berkeley National Laboratory, Berkeley, CA 94720, USA; 4 Department of Chemical Engineering, University of California, Berkeley, CA 94720, USA

## Abstract

**Background:**

Increasing energy costs and environmental concerns have motivated engineering microbes for the production of "second generation" biofuels that have better properties than ethanol.

**Results and conclusion:**

*Saccharomyces cerevisiae *was engineered with an n-butanol biosynthetic pathway, in which isozymes from a number of different organisms (*S. cerevisiae*, *Escherichia coli*, *Clostridium beijerinckii*, and *Ralstonia eutropha*) were substituted for the Clostridial enzymes and their effect on n-butanol production was compared. By choosing the appropriate isozymes, we were able to improve production of n-butanol ten-fold to 2.5 mg/L. The most productive strains harbored the *C. beijerinckii *3-hydroxybutyryl-CoA dehydrogenase, which uses NADH as a co-factor, rather than the *R. eutropha *isozyme, which uses NADPH, and the acetoacetyl-CoA transferase from *S. cerevisiae *or *E. coli *rather than that from *R. eutropha*. Surprisingly, expression of the genes encoding the butyryl-CoA dehydrogenase from *C. beijerinckii *(*bcd *and *etfAB*) did not improve butanol production significantly as previously reported in *E. coli*. Using metabolite analysis, we were able to determine which steps in the n-butanol biosynthetic pathway were the most problematic and ripe for future improvement.

## Background

Soaring energy costs and increased awareness of global warming have motivated production of renewable, biomass-derived fuels and chemicals. The reasons for producing alternatives to ethanol, the current biofuel standard, are numerous and clear: ethanol suffers from low energy density, it is hydroscopic, it cannot be piped, and it is costly to distill, an aspect that detracts from the total energy output of its production. Ideally, biofuels will require minimal energy to separate from fermentation broths, be non-toxic to the host micro-organism, and be efficiently produced from a variety of feedstocks [1]. Compared to ethanol, n-butanol is more hydrophobic, has a higher energy density, can be transported through existing pipeline infrastructure, and can be mixed with gasoline at any ratio. Thus, n-butanol is a substantially better biofuel than ethanol.

n-Butanol can be produced either chemically from petroleum or fermentatively in a variety of Clostridial species. Advances in biotechnology and increased petroleum costs have renewed interest in fermentative n-butanol production, however, Clostridia are not ideal because of the relative lack of genetic tools to manipulate their metabolism, their slow growth, their intolerance to n-butanol above 1–2% and oxygen, and their production of butyrate, acetone, and ethanol as byproducts. Thus, there is interest in producing n-butanol in a more suitable industrial organism. Recently, two groups have re-constructed the n-butanol pathway from Clostridia and measured n-butanol production in *Escherichia coli *(~1 mM) [2,3]. We chose *Saccharomyces cerevisiae *as a host for n-butanol production because it is a genetically tractable, well-characterized organism, the current industrial strain alcohol (ethanol) producer, and it has been previously manipulated to produce other heterologous metabolites [4]. Since n-butanol and ethanol only differ by two carbons, *S. cerevisiae *may be able to tolerate high concentrations of n-butanol by the same mechanisms it tolerates ethanol. Recently, S. cerevisiae has been demonstrated to have tolerance to n-butanol [5]. Here we demonstrate the engineering of *S. cerevisiae *for the production of n-butanol and provide insight for the next steps in engineering by detection of intermediate pathway metabolites.

## Results and discussion

### Expression of n-butanol pathway isozymes from a range of organisms in *S. cerevisiae *results in n-butanol production

**Figure 1:**
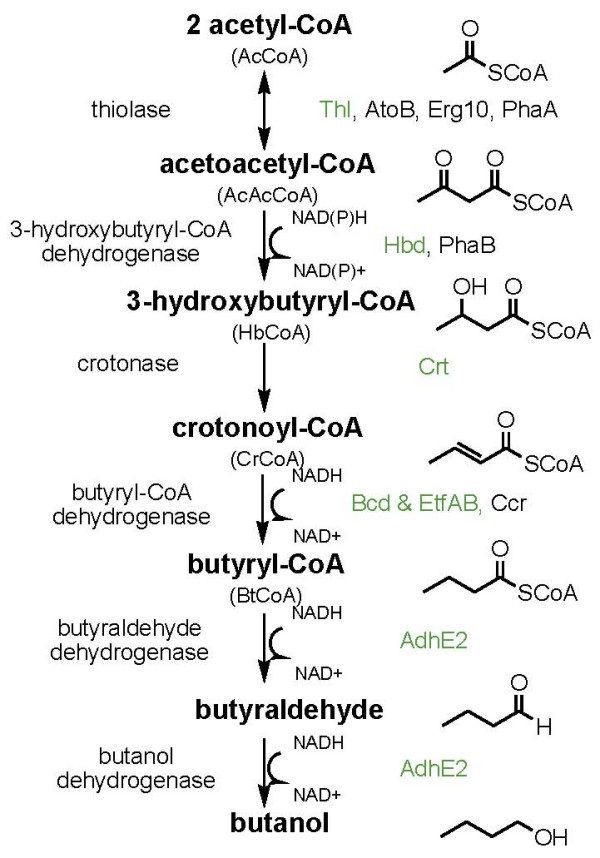
**The n-butanol biosynthetic pathway. The enzymes in green are from *Clostridium beijerinckii*.** Enzymes in black are from other organisms: AtoB, *Escherichia coli*; Erg10, *S. cerevisiae*; PhaA, *Ralstonia eutropha*; PhaB, *Ralstonia eutropha*; Ccr, *Streptomyces collinus*. Each enzyme candidate was evaluated in the pathway for n-butanol production (except *thl*, which is native to Clostridia).

**Figure 2:**
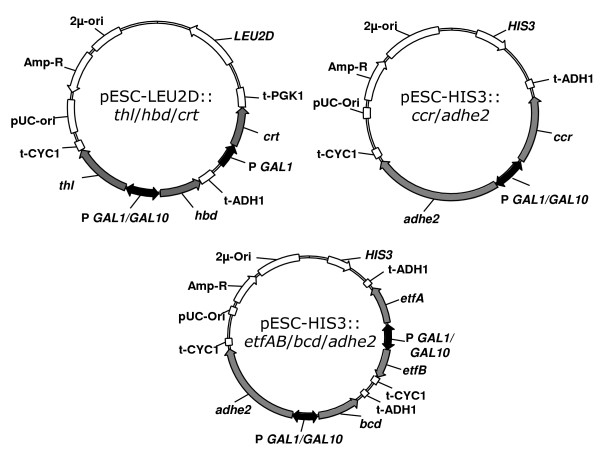
**Representative plasmids used in this study. **Plasmids were constructed by the SLIC method, previously described. They contain the 2μ origin of replication, *LEU2D *or *HIS3 *genes for selection, the *GAL1 *or *GAL10 *promoters, and the *CYC1*, *ADH1*, or *PGK1 *transcription terminators. The first three genes of the n-butanol pathway were placed on the pESC-LEU2D plasmid and the last two or four (in the case of the *etfAB, bcd *bearing strain) genes were placed on the pESC-HIS3 plasmid.

The reactions and corresponding enzymes for n-butanol production are outlined in Figure 1. As enzymes with the same catalytic function from different organisms have different catalytic activity, ability to be expressed, solubility, etc., we sought to test a number of candidates for each reaction in the pathway. The genes encoding these enzymes were cloned into two different plasmids (Figure 2 and Table 1) and transformed into *S. cerevisiae *BY4742.

**Table 1:** Strains used in this study

Name	Genotype	Reference
BY4742	Mat α; *HIS3Δ1; LEU2Δ0; LYS2Δ0; URA3Δ0; YDR242w::kanMX4*	[11]
ESY2	BY4742: pESC-*phaA-phaB-crt-LEU2D + pESC-ccr-adhe2-HIS*	This study
ESY3	BY4742: pESC-*atoB-phaB-crt-LEU2D *+ pESC-*ccr-adhe2-HIS*	This study
ESY4	BY4742: pESC-*ERG10-phaB-crt-LEU2D *+ pESC-*ccr-adhe2-HIS*	This study
ESY5	BY4742: pESC-*phaA-hbd-crt-LEU2D *+ pESC-*ccr-adhe2-HIS*	This study
ESY6	BY4742: pESC-*atoB-hbd-crt-LEU2D *+ pESC-*ccr-adhe2-HIS*	This study
ESY7	BY4742: pESC-*ERG10-hbd-crt-LEU2D *+ pESC-*ccr-adhe2-HIS*	This study
ESY11	BY4742: pESC-*ERG10-hbd-crt-LEU2D *+ pESC-*etfA-etfB-bcd-adhe2-HIS*	This study

**Figure 3:**
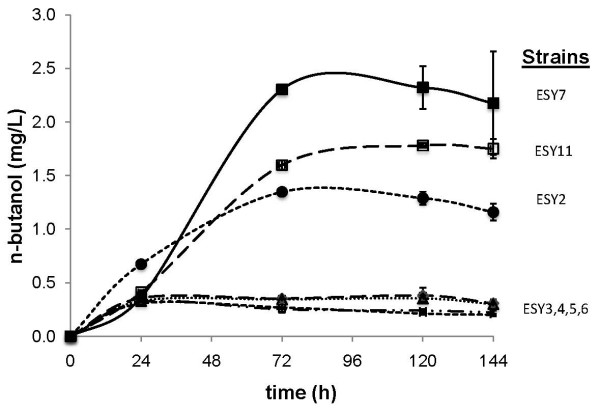
**n-Butanol production from engineered *S. cerevisiae*. **Symbols and strains: black squares, ESY7; empty squares, ESY11; black circles, ESY2; the rest of the samples all produced approximately the same amount of n-butanol and are indicated on the graph. Symbols and error bars represent the mean and standard deviation of triplicate cultures.

The first set of strains, ESY2, ESY3, and ESY4, were engineered with enzymes from *Ralstonia eutropha *(*phaA *and *phaB*), *Streptomyces collinus *(*ccr*), *Clostridium beijerinckii *(*crt *and *adhe2*), *E. coli *(*atoB*), and *S. cerevisiae *(*ERG10*) and only varied in the first committed step, the thiolase (PhaA, AtoB, and ERG10). We tested the enzymes from *R. eutropha*, because these enzymes have previously been demonstrated to retain high activity for the production of polyhydroxyalkanoates in *E. coli *[6]. The two other thiolases were tested because ERG10 is the native thiolase in *S. cerevisiae*, and AtoB has been successfully used to overproduce acetoacetyl-CoA (AcAcCoA) in other metabolic pathways [7]. These strains produced different levels of n-butanol (Figure 3), the highest being ESY2 (1 mg/L), suggesting that PhaA is the best thiolase in this specific pathway configuration. From these strains, we constructed and tested strains with different enzyme candidates for their ability to increase n-butanol production (Table 1 and Figure 3).

The second set of strains was constructed to compare n-butanol production using different isozymes for 3-hydroxybutyryl-CoA (HbCoA) dehydrogenase, which converts AcAcCoA into HbCoA. One of these isozymes uses NADPH (PhaB) as a cofactor, whereas the other isozyme uses NADH (Hbd) as a cofactor. As cells growing under fermentative conditions generally have excess NADH, Hbd might be preferred to PhaB [2]. Although PhaA appeared to be the best thiolase based on the results using the previous set of strains, it was unclear that PhaA would function as well with HbCoA dehydrogenase as it did with PhaB (given the context dependence of some pathway configurations [8]). Thus, we tested Hbd with all three thiolases. These strains, ESY5, ESY6, and ESY7, all produced detectable levels of n-butanol, while ESY7 (*ERG10*, *hbd*) doubled the production of n-butanol over the previously highest producing strain, ESY2 (*phaA*, *phaB*), reaching 2.5 mg/L. It is difficult to attribute the increase in production to one specific enzyme, because ESY7 harbors a different thiolase (*ERG10*) and a different HbCoA dehydrogenase (*hbd*) that utilizes NADH, compared to ESY2. It is curious why the strain harboring PhaA is not as productive with Hbd as it is with PhaB. It may be that PhaA and PhaB have been optimized through evolution to work together to maximize production of polyhydroxybutyrate in *R. eutropha*. That the strain harboring ERG10 and Hbd is the highest producer is not surprising given that ERG10 is the native thiolase and Hbd uses NADH, which should be in ample supply under fermentation conditions.

The final strain (ESY11) was made to determine if an alternative butyryl-CoA (BtCoA) dehydrogenase, which was previously shown to greatly increase n-butanol production in *E. coli *[2], would improve n-butanol production over that by ESY7. Surprisingly, expression of the *C. beijerinckii bcd *and *etfAB *(ESY11) did not improve n-butanol production significantly. Notably, ESY11 was the second highest n-butanol producer and compared to ESY7, only differed in the BtCoA dehydrogenase.

In summary, the best strain, ESY7, produced 2.5 mg/L n-butanol from 2% galactose as a carbon source. This strain overexpressed the native thiolase (ERG10) and an HbCoA dehydrogenase (Hbd) that utilized NADH, which the cell should have in plentiful supply under fermentative conditions. ESY7, which also harbored *ccr*, produced slightly more n-butanol than ESY11, which harbored *bcd *and *etfAB*.

### Analysis of intermediary metabolites

**Figure 4:**
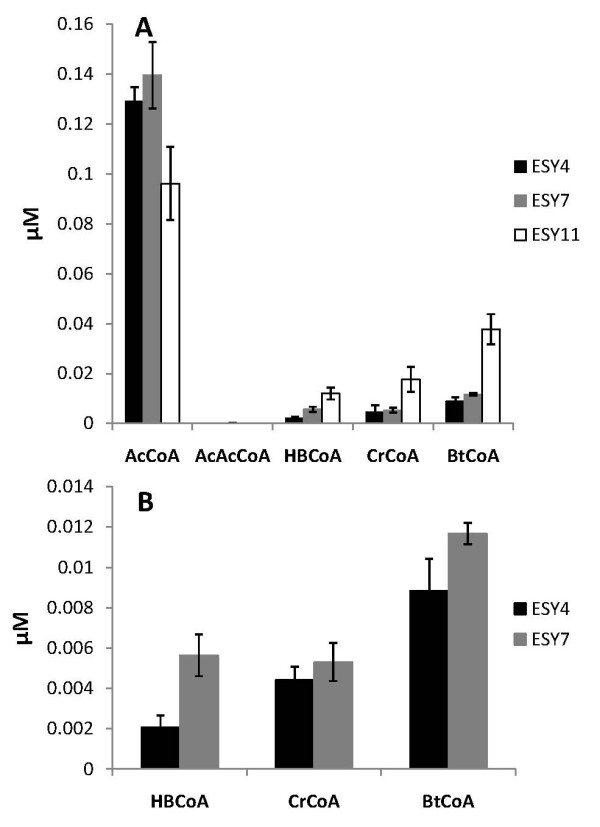
**n-Butanol pathway intermediates at 24 h.** Bars and strains: black bars, ESY4; gray bars, ESY7; white bars, ESY11. (A) All pathway intermediates in strains ESY4, 7 and 11. (B) HbCoA, CrCoA and BtCoA intermediates in strains ESY4 and ESY7. Levels of AcCoA were similar except for strains ESY11 (A). Levels of 3-hydroxybutyryl-CoA (HbCoA) and butyryl-CoA (BtCoA) were notably higher in ESY7 compared to ESY4, while crotonyl-CoA (CrCoA) was relatively similar in the two strains. Values and error bars represent the mean and standard deviation of triplicate cultures.

In an attempt to explain the differences in n-butanol production by the various strains, we analyzed the pathway intermediates. Previously, this analysis has proven to be successful in interrogating and optimizing metabolic pathways [9]. We developed a single LC-MS method to monitor all of the metabolites in the n-butanol biosynthetic pathway simultaneously. Detection of all the metabolic standards was successful, while detection of intermediates in cell extracts was successful for all intermediates except AcAcCoA. Although we analyzed the metabolic pathway intermediate levels in all strains, we present the results for strains ESY4, ESY7, and ESY11 only. We chose ESY7 because it was the highest producer, ESY4 because it differed from ESY7 only in the HbCoA dehydrogenase (PhaB in ESY4 versus Hbd in ESY7) and yet displayed a 10-fold difference in n-butanol titers (Figure 4). We chose ESY11 because it was the second highest producer and differed from ESY7 only in the choice of BtCoA dehydrogenase (Ccr in ESY7 and EtfAB/Bcd in ESY11).

At 24 h, the levels of AcCoA in strains ESY4 and ESY7 were indistinguishable, while the levels of HbCoA and BtCoA were higher in strain ESY7 than in ESY4, suggesting that the Hbd enzymatic reaction had higher flux than the PhaB enzymatic reaction. ESY11, which differs from ESY7 only in the BtCoA dehydrogenase (Ccr in ESY7 versus EtfAB/Bcd in ESY11), accumulated BtCoA, which suggests a bottleneck at BtCoA. Increased expression of *adhe2 *in ESY11 could alleviate the accumulation of BtCoA and improve the production of n-butanol, perhaps beyond that of ESY7. Since the levels of the other pathway metabolites were essentially indistinguishable between ESY4 and ESY7, we conclude that production of HbCoA by 3-hydroxybutyryl-CoA dehydrogenase is the rate-limiting reaction that determines the n-butanol production in this context. Proteomics analysis showed that Hbd (in ESY7) and PhaB (in ESY4) were expressed at equivalent levels (data not shown). As such, the differences in the NADPH and NADH levels may explain the higher production of n-butanol by ESY7 relative to ESY4. Indeed, NADPH limitation has been demonstrated for other pathways [10]. Additionally, there is slightly more BtCoA in ESY7 than in ESY4, which may ultimately correspond to higher n-butanol production by ESY7. Interestingly, BtCoA seems to accumulate in all strains and suggests further engineering of Adhe2 is necessary. In support of this claim, we analyzed the solubility of Adhe2 by western blot and found the majority of the protein in the insoluble fraction (data not shown). The trends in the levels of pathway intermediates between strains ESY7 and ESY4 were maintained through 72 h. Furthermore, increased expression of *crt *may alleviate the higher levels of HbCoA and create a more balanced pathway.

## Conclusion

Here we provide the first demonstration of n-butanol production in *S. cerevisiae *to 2.5 mg/L and tested a variety of isozymes for different reactions in the metabolic pathway. There are a number of obstacles to overcome when expressing heterologous biosynthetic pathways, including enzyme choice, verification of gene expression, balancing of metabolic pathway intermediates and protein levels, and removal of competing pathways, all the while increasing production and maintaining host organism viability. We successfully demonstrated substitution of isozymes and analysis of pathway intermediates and determined potential engineering targets for increasing n-butanol biosynthesis. Comparison of our strain to the native n-butanol producers, Clostridia (~10 g/L), or the recently engineered *E. coli *strains (~0.5 g/L) provides a goal for future n-butanol titer. Given the results presented above and *S. cerevisiae*'s other attributes–inherent tolerance to solvents [5], widespread use for industrial production of ethanol, and ability to withstand oxygen (as opposed to Clostridia)–*S. cerevisiae *may be an ideal host for industrial n-butanol production. While increases in product titer will certainly be necessary, increases of this magnitude and greater have precedence [4].

## Methods

### Chemicals

Ethyl acetate was purchased from Sigma-Aldrich (St. Louis, MO). Complete Supplement Mixtures for formulation of Synthetic Defined (SD) media were purchased from Qbiogene (Irvine, CA). All other media components were purchased from Sigma-Aldrich.

### Strains and media

*Clostridium beijerinckii *NCIMB 8052 was purchased from ATCC, catalog number 51743. *E. coli *strains DH10B and DH5á were used for bacterial transformation and plasmid amplification in the construction of the expression plasmids used in this study. The strains were cultivated at 37°C in Luria-Bertani medium with 100 mg L^-1 ^ampicillin. *S. cerevisiae *strain BY4742 [11], a derivative of S288C, was used as the parent strain for all yeast strains. This strain was grown in rich YPD medium at 30°C [12]. Engineered yeast strains were grown in SD medium [12] with leucine, uracil, histidine, and/or methionine dropped out where appropriate. For induction of genes expressed from the *GAL1 *and *GAL10 *promoters, *S. cerevisiae *strains were grown in 2% galactose as the sole carbon source unless otherwise indicated.

### Plasmid construction

*C. beijerinckii *genes were cloned from genomic DNA: *hbd *encodes 3-hydroxybutyryl-CoA dehydrogenase; *crt*, crotonase; *bcd*, butyryl-CoA dehydrogenase; and *etfA *&*etfB*, two-electron transferring flavoproteins A & B. *phaA *and *phaB *(*Ralstonia eutropha*), *adhe2 *(*C. beijerinckii*), and *ccr *(*Streptomyces collinus*) were synthesized (Epoch Biolabs). All genes were PCR amplified with Phusion polymerase (New England Biolabs). Primers were designed to have 30-bp flanking regions homologous to the plasmid insertion regions, either the *gal1 *or *gal10 *promoter and the *CYC1*, *ADH1*, or *PGK1 *terminator. Plasmid construction was carried out using the Sequence and Ligation Independent Cloning (SLIC) method, previously described [13]. The constructed plasmids were derived from p*ADS*-*AMO*-*CPR*-opt-*LEU2D *plasmid [14] and pESC-*HIS *(Stratagene).

### Yeast transformation and strain construction

Transformation of all *S. cerevisiae *strains was performed using the lithium acetate method [15]. Strains ESY1-11 were constructed by the co-transformation of the indicated plasmids followed by selection on SD-LEU or SD-LEU-HIS plates as appropriate.

### Yeast cultivation

All optical density measurements at 600 nm (OD_600_) were taken using a Beckman DU-640 spectrophotometer. To measure n-butanol production, culture tubes containing 5 mL of SD (2% galactose) medium (with appropriate amino acid omissions as described above) were inoculated with the strains of interest. These innocula were grown at 30°C to an OD_600 _between 1 and 2. Capped serum vials (100 mL) containing 50 mL SD medium were inoculated to an OD_600 _0.05 with these seed cultures in order to achieve a "semi" anaerobic condition. Samples were collected at 24, 72, 120, and 144 h and analyzed for metabolites as discussed below.

### Metabolite detection

For metabolite analysis, cultures were sampled (10 mL) at 24, 72, 120, and 144 h. For n-butanol detection, 2 mL ethyl acetate containing n-pentanol (0.005% v/v), an internal standard, was added to the 10 mL sample and vortexed for 1 min. The ethyl acetate was then recovered and applied to a Thermo Trace Ultra gas chromatograph (GC) equipped with a Triplus AS autosampler and a TR-WAXMS column (Thermo Scientific). The samples were run on the GC with the following program: initial temperature, 40°C for 1.2 min, ramped to 130°C at 25°C/min, ramped to 220°C at 35°C/min. Final quantification analysis was achieved with Xcalibur software. For pathway intermediate analysis, a method previously established was employed [16]. Specifically, 10-mL samples were pelleted (6000 rpm, 5 min, 4°C). The supernatant was aspirated, and the cells were suspended in 1 mL of 10% TCA containing propionyl-CoA (10 μM) as an internal standard. The cells were bead-beaten for 4 min. The supernatant was collected and neutralized with 2× volume of 1 M octylamine. Samples were then filtered and separated on a Zorbax 300SB-C1 8 column (Agilent; 2.1 mm i.d. × 10 cm length) using an Agilent 1100 series HPLC at a flow rate of 150 μL/min. The LC conditions used were adapted from Pitera *et al*. [9] Briefly, samples were run from initial conditions of *t *= 0 min in 95% 100 mM ammonium acetate (Buffer A), 5% 100 mM ammonium acetate:acetonitrile (70%:30%) (Buffer B); *t *= 5 min, 95% Buffer A, 5% Buffer B; *t *= 12 min, 80% Buffer A, 20% Buffer B; *t *= 16 min, 10% Buffer A, 90% Buffer B; *t *= 25 min, 10% Buffer A, 90% Buffer B; *t *= 28 min, 95% Buffer A, 5% Buffer B; *t *= 45 min, 95% Buffer A, 5% Buffer B. The LC system was interfaced to an Applied Biosystems Q TRAP 2000 LC/MS/MS via a Turbo Ionspray source operating in the positive ion mode (5500 V). The MS was operated in single-ion-monitoring (SIM) mode with a dwell time of 200 ms for each CoA metabolite of interest. Data were collected and analyzed with Analyst™ 1.4.2 (Applied Biosystems).

## Competing interests

JDK has financial interests in Amyris and LS9, both of which are involved in producing advanced biofuels.

## Authors' contributions

EJS conceived and designed the study with the input of JDK. EJS and RC constructed the plasmids and strains. EJS, RC, and NP carried out the intermediate metabolite and production experiments. EJS, SM, AR, and CP performed protein analyses. EJS drafted the manuscript. All authors have critiqued and approved the final manuscript.
